# Influenza vaccine effectiveness in reducing severe outcomes over six influenza seasons, a case-case analysis, Spain, 2010/11 to 2015/16

**DOI:** 10.2807/1560-7917.ES.2018.23.43.1700732

**Published:** 2018-10-25

**Authors:** Pere Godoy, Arantxa Romero, Núria Soldevila, Nuria Torner, Mireia Jané, Ana Martínez, Joan A Caylà, Cristina Rius, Angela Domínguez, The Working Group on Surveillance of Severe Influenza Hospitalized Cases in Catalonia

**Affiliations:** 1 Agència de Salut Pública de Catalunya, Barcelona, Spain; 2 CIBER Epidemiología y Salud Pública, Barcelona, Spain; 3 IRBLleida. Institut de Recerca Biomèdica de Lleida, Lleida, Spain; 4 Universitat de Barcelona, Barcelona, Spain; 5 TB Research Unit Foundation (fuiTB), Barcelona, Spain; 6 Agència de Salut Pública de Barcelona, Barcelona, Spain; 7 The Working Group on Surveillance of Severe Influenza Hospitalized Cases in Catalonia have been listed at the end of this article

**Keywords:** influenza, vaccine effectiveness, disease severity, hospitalised patients, Spain

## Abstract

**Introduction:**

When influenza vaccination is ineffective in preventing influenza virus infection, it may still reduce the severity of influenza-associated disease. Here, we estimate the effect of influenza vaccination in preventing severe outcomes e.g. intensive care unit (ICU) admission and death, even though it did not prevent influenza virus infection and subsequent hospitalisation.

**Methods:**

An observational case–case epidemiological study was carried out in 12 sentinel hospitals in Catalonia (Spain) over six influenza seasons 2010/11–2015/16. Cases were individuals with severe laboratory-confirmed influenza virus infection and aged 18 years and older. For each reported case we collected demographic, virological and clinical characteristics. Logistic regression was used to estimate the crude, adjusted odd ratios (aOR) and 95% confidence intervals (CI).

**Results:**

Of 1,727 hospitalised patients included in the study, 799 were female (46.7%), 591 (34.2%) were admitted to the ICU and 223 (12.9%) died. Influenza vaccination uptake was lower in cases that required ICU admission or died (21.2% vs 29.7%, p < 0.001). The adjusted influenza vaccination effectiveness in preventing ICU admission or death was 23% (95% CI: 1 to 40). In an analysis restricted to sex, age group and antiviral treatment, influenza vaccination had a positive effect on disease severity in all age groups and categories.

**Conclusions:**

We found that influenza vaccination reduced the severity of disease even in cases where it did not prevent infection and influenza-associated hospitalisation. Therefore, increased vaccination uptake may reduce complications, ICU admission and death.

## Introduction

Each year, 5–20% of the global population are infected by the influenza virus, which is estimated to result in 3–5 million cases of severe illness and 300,000 to 500,000 deaths worldwide [1].

Influenza surveillance is essential to determine the timing and spread of influenza and trace variations in circulating influenza viruses to provide information on the composition of the seasonal influenza vaccine [2]. Sentinel surveillance of patients hospitalised due to severe laboratory-confirmed influenza has shown that influenza is an important cause of severe illness and death, mainly among those aged 65 years and older and patients with underlying chronic diseases [3].

The influenza vaccine is the best tool for the prevention of influenza and its complications, particularly in patients with underlying chronic diseases and those aged 65 years and older [4-7]. Influenza vaccination is effective in preventing primary healthcare visits and hospital admissions for laboratory-confirmed influenza [8], but chronic conditions and other risk factors, including older ages, may interfere with and hinder a successful vaccine response [9,10].

When influenza vaccination is ineffective in preventing influenza virus infection it may still have an additional effect by reducing the severity of influenza [11]. It has been suggested that while protection against influenza virus infection is primarily mediated through the antibody response, protection against severe outcomes is mediated through cellular immune responses affecting viral clearance [4,11]; however, the effect of influenza vaccination on influenza severity remains uncertain. While some studies evaluating hospital admission in patients with laboratory-confirmed influenza have failed to demonstrate a protective effect of influenza vaccination [12,13], others have found it has an effect on reducing influenza severity [14]. Case–case studies are considered the best approach to estimate a net effect of influenza vaccination on the post-infection outcomes only in subjects who become infected [15].

In 2010, Catalonia, a region in the north-east of Spain with 7.5 million inhabitants, initiated surveillance of patients hospitalised due to laboratory-confirmed influenza with the aim of (i) estimating the severity of seasonal influenza epidemics according to the characteristics of the influenza virus, (ii) identifying risk groups for severity and, (iii) providing information to improve influenza prevention and control. The system includes 12 hospitals covering 4,644,543 persons (62% of the entire population) [16]. The surveillance system reports on patients hospitalised with severe laboratory-confirmed influenza admitted to one of these hospitals every influenza season since 2010/11. The hospital-based surveillance was set up to supplement the information provided by the influenza sentinel surveillance system based on surveillance information from primary healthcare network physicians [16].

The presented study aimed to estimate the effect of influenza vaccination during influenza seasons 2010/11–2015/16 in preventing severe outcomes, e.g. intensive care unit (ICU) admission and death, when the influenza vaccination did not prevent influenza and subsequent hospitalisation.

## Methods

We conducted a hospital-based observational case–case epidemiological study on the effect of influenza vaccination in reducing disease severity in patients hospitalised due to laboratory-confirmed influenza over six influenza seasons (2010/11–2015/16).

### Study population and data collected

The study population were all reported cases that were those 18 years and older that had been hospitalised with severe laboratory-confirmed influenza virus infection in the 12 sentinel hospitals in Catalonia during six influenza seasons (2010/11–2015/16).

We included patients that had been hospitalised for more than 24 hours in any of the 12 participating hospitals due to influenza-like-illness (ILI) with symptom onset more than 7 days before admittance. Definition for ILI based on the European Union case definition [17] can be seen in Box.

Box **Influenza-like-illness case definition, Catalonia, Spain, influenza seasons 2010/11—2015/16**

Influenza-like-illness (ILI) was defined as a combination of the following three criteria:

(i) Sudden symptom onset:

(ii) ≥ 1 of the following symptoms:

• fever (≥ 38 °C)

• headache

• myalgia or malaise

and

(iii) ≥ 1 of the following respiratory symptoms:

• cough

• sore throat or

• shortness of breath (dyspnea).

Patients were recruited in one of the participating hospitals by a sentinel physician who screened all patients admitted overnight presenting with severe ILI. All patients had a nasopharyngeal or throat swab (bronchoalveolar lavage fluid or tracheal aspirate for ICU patients) and influenza virus infection was detected using reverse transcription polymerase chain reaction (RT-PCR).

Public health officers from the surveillance units in Catalonia, collected data from each reported case by interview using a structured questionnaire from the epidemiological surveillance network. Information included socio-demographic data, obesity (body mass index (BMI) > 40), pregnancy, major chronic conditions (e.g. asthma, chronic obstructive pulmonary disease (COPD), chronic renal disease, immunodeficiency (HIV infection or other), chronic cardiovascular disease, chronic liver disease).

In addition, information on the laboratory diagnosis, symptom onset, hospital admission and discharge dates, complications (primary or secondary pneumonia with or without bacterial coinfection, respiratory distress syndrome or multiple organ failure), antiviral treatment, influenza vaccination status and date of administration, ICU admission, and death were collected from medical records for all reported cases.

### Laboratory data

Patient samples were first tested for type of virus in the laboratories of the participating hospitals using an in-house real-time influenza A and B PCR after manual nucleic acid extraction. Amplification was performed in an ABI 7500 thermocycler. All samples, including those with unsubtyped influenza virus, were sent to the Catalan Influenza Reference Centre (Hospital Clinic, Barcelona, Spain) to determine the subtype. Samples found to be positive in the reference centre were molecularly subtyped for viruses known to be circulating at the time, namely type A (subtypes H1N1pdm09 and H3N2) and type B. Molecular subtyping was used to determine the H subtype for influenza A and the lineage for influenza B. Subtyping failed in some cases due to a low viral load and such samples were classified as ‘unidentifiable’. .

### Statistical analysis

We compared hospitalised cases of severe influenza who required ICU admission or those who died at the hospital after being admitted, with hospitalised cases of severe influenza that did not require ICU admission and did not die. We compared baseline characteristics and vaccination status between the two groups. The baseline variables considered were: sex, age (18–64 years, and those aged 65 years and older), virus (sub)types, major chronic conditions, pregnancy, complications and antiviral treatment. The chi-squared test and Fisher’s exact test were used for categorical variables and the t-test for continuous variables for comparisons between the groups.

To investigate relationships between the dependent variables (ICU admission or death) and the independent variables studied (including influenza vaccination), a case–case bivariate analysis was conducted. Possible interactions between influenza vaccination status and any independent variable were analysed by logistic regression. Independent variables were checked for collinearity using the variance inflation factor (Katz).

The effectiveness of influenza vaccination was calculated using the formula vaccine effectiveness (VE) = (1 − odds ratio) x 100. Logistic regression was used to estimate the crude and adjusted odds ratios (aOR) and their corresponding 95% confidence intervals (CI). A multivariate analysis was performed using the change-in-estimate criterion, including potential confounders in the model when the OR changed by ≥ 10%. Since influenza vaccination rates among those aged 65 years and older were high and increased substantially with age, to account for dissimilar distributions of baseline characteristics between vaccinated and unvaccinated groups and to reduce confounding, we predicted the probability of influenza vaccination using the propensity score method. The propensity score was estimated by logistic regression with seasonal influenza vaccination as the outcome and age, sex, comorbidities, pregnancy, type of virus and antiviral treatment as independent variables. The propensity score was used as a continuous covariate in a final logistic regression model.

To assess the robustness of the estimate, influenza VE was also calculated in patient subgroups according to sex, age and antiviral treatment. The analysis was performed using the SPSS v.24 statistical package and the R v3.3.0 statistical software [18].

## Results

A total of 1,727 patients aged 18 years and older hospitalised with severe laboratory-confirmed influenza were Included in the study, of which 736 (43.0%) were female, 799 (46.7%) were aged 18–64 years and 912 (53.3%) were aged 65 years or older. In the six seasons studied (2010/11–2015/16), 1,470 (85.9%) patients were infected with influenza A viruses (H_1_N_1_ = 572 and H_3_N_2_ = 572) and 241 (14.1%) with influenza B viruses. Of the 1,727 patients, 591 (34.2%) were admitted to the ICU and 223 (12.9%) died. A total of 1,285 (74.4%) patients presented with one or more influenza risk factors, but only 450 (26.1%) had received the influenza vaccine. Influenza vaccination uptake was similar in males and females (27.1% vs 25.3%) but higher in those aged 65 years or older compared with those aged 18–64 years (41.6% vs 8.9%). There were 26 pregnant women, none of which were vaccinated (Table 1).

**Table 1:** Characteristics of patients hospitalised due to laboratory-confirmed influenza, Catalonia, influenza seasons 2010/11–2015/16 (n = 1,711)

Characteristics	Total		Vaccinated		Unvaccinated		p value
	n	%	n	%	n	%	
All cases	1,711	100	450	100	1,261	100	NA
**Sex**							
Male	975	57	264	58.7	711	56.4	0.401
Female	736	43	186	41.3	550	43.6	
**Age**							
18–64	799	46.7	71	15.8	728	57.7	< 0.001
≥ 65	912	53.3	379	84.2	533	42.3	
**Type of influenza virus**							
A	1,470	85.9	375	83.3	1,095	86.8	0.067
B	241	14.1	75	16.7	166	13.2	
**Comorbidities**							
Obesity (BMI > 40)	181	10.6	50	11.1	131	10.4	0.669
COPD	441	25.8	159	35.3	282	22.4	< 0.001
Diabetes mellitus	425	24.8	156	34.6	269	21.3	< 0.001
Chronic renal disease	235	13.7	94	28.9	141	11.2	< 0.001
Immunodeficiency	333	19.5	74	16.4	259	20.5	0.060
Chronic cardiovascular disease	504	29.5	202	44.9	302	23.9	< 0.001
Chronic liver disease	112	6.5	21	4.7	91	7.2	0.063
**Complications**							
Pneumonia	1,277	74.9	316	70.2	961	76.2	0.016
ARD	1,019	60.8	144	32.0	513	40.7	0.001
Multi-organ failure	176	10.6	40	8.9	136	10.8	0.270
ICU admission	591	34.5	108	24.0	483	38.3	< 0.001
Death	223	13.0	63	14.0	160	12.7	1.533
Pregnancy^a^	26	1.5	0	0.0	26	2.1	NC

ARD: Acute respiratory distress; BMI: body mass index; COPD: chronic obstructive pulmonary disease; ICU: intensive care unit; NA: not applicable; NC: not calculable.

^a^Pregnancy was not considered a complication and was in another category.

There were differences between patients admitted to the ICU or who died and those who did not. Patients who required ICU admission or died were more frequently male (60.8% vs 54.4%, p < 0.001), aged 18–64 years (55.8% vs 40.8%, p < 0.001), obese (12.6% vs 9.2%, p = 0.02), immunodeficient (23.0% vs 17.1% p < 0.001), had chronic liver disease (8.4% vs 5.5% p = 0.01), were pregnant (2.3% vs 1% p = 0.03) and a higher proportion stayed in hospital more than 14 days (51.4% vs 17.0%, p < 0.001), but there were no differences in vaccinated patients vs unvaccinated patients in the average length of hospital stay (14.11 ± 15.58 and 15.01 ± 16.16 days, respectively; p = 0.31), the type of virus or whether antiviral treatment was administered before or after 48 hours after hospital admission (Table 2).

**Table 2:** Factors associated with intensive care unit admission and death, Catalonia, influenza seasons 2010/11–2015/16 (n = 1,727)

Characteristics	ICU/death (n = 692)		Non-ICU/death (n = 1,035)		Crude OR	95% CI	p value
	n	%	n	%			
**Seasonal vaccination**							
Yes	146	21.3	304	29.7	0.64	(0.51 to 0.80)	< 0.001
No	540	78.7	721	70.3	Ref		
**Age**							
18–64 years	386	55.8	422	40.8	Ref		
≥ 65 years	306	44.2	613	59.2	0.55	(0.45 to 0.66)	< 0.001
**Sex**							
Male	271	39.2	472	45.6	0.77	(0.63 to 0.93)	0.008
Female	421	60.8	563	54.4	Ref		
**Type of virus**							
A	602	87	883	85.3	1.15	(0.87 to 1.52)	0.325
B	90	13	152	14.7	Ref		
**COPD**							
Yes	187	27.0	255	24.6	1.13	(0.91 to 1.41)	0.27
No	505	73.0	780	75.4	Ref		
**Obesity**							
Yes	87	12.6	95	9.2	1.42	(1.04 to 1.94)	0.02
No	605	87.4	940	90.8	Ref		
**Diabetes mellitus**							
Yes	170	24.6	261	25.2	0.97	(0.77 to 1.21)	0.76
No	522	75.4	774	74.8	Ref		
**Chronic renal disease**							
Yes	100	14.5	136	13.1	1.12	(0.84 to 1.47)	0.44
No	592	85.5	899	86.9	Ref		
**Immunodeficiency**							
Yes	159	23.0	177	17.1	1.45	(1.14 to 1.84)	0.003
No	533	77.0	858	82.9	Ref		
**Chronic cardiovascular disease**							
Yes	196	28.3	312	30.1	0.92	(0.74 to 1.13)	0.42
No	496	71.7	723	69.9	Ref		
**Chronic liver disease**							
Yes	58	8.4	55	5.3	1.63	(1.11 to 2.39)	0.01
No	634	91.6	980	94.7	Ref		
**Pregnancy**							
Yes	16	2.3	10	1.0	2.43	(1.09 to 5.38)	0.03
No	676	97.7	1025	99.0	Ref		
**NI treatment**							
Yes	635	91.8	942	91.0	1.10	(0.78 to 1.55)	0.59
≤ 48h symptom onset	154	23.1	283	28.4	0.89	(0.60 to 1.30)	0.54
> 48h symptom onset	455	68.3	619	62.2	1.20	(0.84 to 1.70)	0.31
No	57	8.6	93	9.3	Ref		
**Number of risk factors**							
0	172	24.9	270	26.1	0.78	(0.58 to 1.06)	0.11
1	241	34.8	378	36.5	0.78	(0.59 to 1.04)	0.09
2	155	22.4	235	22.7	0.81	(0.62 to 1.10)	0.18
> 2	124	17.9	152	14.7	Ref		
**Hospital stay**							
0–7 days	145	21	539	52.1	0.24	(0.197 to 0.30)	< 0.001
> 7 days	545	79	145	21	Ref		
**Hospital stay**							
0–14 days	335	48.6	859	83	0.19	(0.15 to 2.41)	< 0.001
> 14 days	355	51.4	176	17	Ref		

CI: confidence interval; COPD: chronic obstructive pulmonary disease; ICU: intensive care unit; NI: Neuraminidase inhibitors; OR: odds ratio; Ref: reference.

Influenza vaccination uptake was lower in patients who required ICU admission or died (146/686: 21.3% vs 304/1025: 29.7%, p < 0.001) (Table 2). The unadjusted influenza VE in preventing ICU admission or death was 36% (95% CI: 20 to 49) and, except in one season (2013/14), had a positive effect in all influenza seasons, although the effect was not significant in some seasons (2010/11, 2011/12, 2012/13 and 2013/14) (data not shown).

In the multivariable regression model, the variables associated with ICU admission or death were in those aged 65 years or older (aOR: 0.56; 95% CI: 0.45 to 0.68) and comorbidities (aOR: 1.36; 95% CI: 1.07 to 1.73). The adjusted effectiveness of influenza vaccination in preventing ICU admission or death was 22% (95% CI: 1 to 39) and remained the same after adjustment by the propensity score (23%; 95% CI: 1 to 40) (Table 3, propensity score data not shown). In the analysis restricted to sex, age group and antiviral treatment, influenza vaccination had a positive effect in all groups and categories, although in females and those aged 65 years and older, the effectiveness was lower and was not significant (Table 4).

**Table 3:** Effect of influenza vaccination in reducing severe outcomes in patients hospitalised due to laboratory-confirmed influenza, Catalonia, Spain, influenza seasons 2010/11–2015/16 (n = 1,727)

Characteristics	ICU/death (n = 692)		Non-ICU/death (n = 1,035)		Adjusted OR	95% CI	p value
	n	%	n	%			
**Seasonal vaccine**							
Yes	146	21.3	304	29.7	0.78	(0.61 to 0.99)	0.048
No	540	78.7	721	70.3	Ref		
**Age**							
18-64 years	386	55.8	422	40.8	Ref		
≥ 65 years	306	44.2	613	59.2	0.56	(0.45 to 0.68)	< 0.001
**Comorbidities**							
Yes	520	75.1	765	73.9	1.36	(1.07 to 1.73)	0.011
No	172	24.9	270	26.1	Ref		

CI: confidence interval; ICU: intensive care unit; OR: odds ratio; Ref: reference.

**Table 4:** Effect of seasonal influenza vaccination in reducing severity among subgroups of patients hospitalised due to laboratory-confirmed influenza, Catalonia, Spain, influenza seasons 2010/11–2015/16 (n = 1727)

Analysis subset	ICU/death	Non-ICU/death	Crude vaccination effect	95% CI	Adjusted vaccination effect (%)	95% CI	p-value
**Sex**							
Female	271	472	27	-5 to 49	3	-43 to 34	0.886
Male	421	563	43	22 to 57	33	8 to 51	0.014
**Age**							
18–64	386	422	6	-53 to 43	13	-42 to 47	0.574
≥ 65	306	613	33	-2 to 42	25	-1 to 43	0.050
**NI treatment**							
Yes	635	942	36	18 to 49	21	-2 to 39	0.070
No	57	93	37	-30 to 70	57	2 to 81	0.031

CI: confidence interval; ICU: intensive care unit; NI: Neuraminidase inhibitors.

## Discussion

This study, based on surveillance of hospitalised cases of severe laboratory-confirmed influenza from 2010/11–2015/16 in Catalonia, showed an effectiveness of influenza vaccination in reducing ICU admission or death of 23%. Most patients presented with more than one influenza risk factor, but only 26.1% had received seasonal influenza vaccination, suggesting an important potential impact of vaccination in reducing influenza severity.

Our results are consistent with other studies, for example, a study conducted in the United States (US) by Catania et al. also found that patients requiring ICU admission had a lower influenza vaccination coverage [19]; A Spanish study by Casado et al. found that influenza vaccination was associated with a reduction in the odds of in-hospital death and ICU admission in adults hospitalised with laboratory-confirmed influenza [20]and a French study by Loubet et al. reported a reduction in the risk of ICU admission but not death in patients hospitalised with laboratory-confirmed influenza [21]. Our results and those of these studies, suggest that influenza vaccination is a factor for a good prognosis, as it reduces influenza-associated disease severity in patients, in whom vaccination did not prevent influenza.

A similar population-based study of patients hospitalised with laboratory-confirmed influenza by Arriola et al. conducted in the US during influenza season 2012/13 [22] reported that 71% of patients were aged 65 years or older, 91% had medical conditions and 55% had been vaccinated. No association was found between influenza vaccination and ICU admission, death, pneumonia, or the length of hospital or ICU stay. However, after matching patients by the vaccination propensity score, they found that the length of ICU stay was reduced by a factor of 0.6 (95%CI: 0.4 to 0.8) among vaccinated 50–64 year olds compared with unvaccinated patients [22]. This is partly in line with our findings, as while we found a shorter length of hospital stay in vaccinated patients than in unvaccinated patients the difference was not statistically significant.

Further, a study by Castilla et al. [23] also looking at hospitalised cases with laboratory-confirmed influenza found that vaccination protected against severe influenza (aOR: 0.42; 95% CI: 0.22 to 0.80) and suggested that vaccination might be more effective in preventing severe than mild illness [23].

The influenza vaccination uptake in those aged 65 years and older in this study (41.6%) was lower than that of the same age group in the Spanish general population (55.5%) [24], and suggests that improving influenza vaccination coverages may have a noteworthy effect in reducing influenza severity.

Two main approaches are currently used to estimate the post-infection effects of vaccination. The first includes all studied individuals whether they become infected or not, and relies on of the following study designs: cohort, case–control or test-negative case–control. This approach enjoys the statistical validity associated with an intent-to-treat analysis and provides an assessment of the overall benefits of vaccination. However, such an approach does not distinguish between vaccine effects on susceptibility to infection and effects on the post infection endpoints of interest.

The second approach, which we used, includes only infected individuals and relies on a case–case study design. It uses the positive infection status to estimate the net effect of the vaccination on the post-infection endpoint. However, individuals that are infected, even though vaccinated, are unlikely to be identical to infected individuals that are unvaccinated and this can lead to biased interpretations. For example, a vaccine might better protect people with strong immune systems, so that infected vaccines tend to have weaker immune systems on average compared with infected unvaccinated cases. As a result, infected vaccinated persons could have worse post-infection outcomes on average than the infected controls due solely to selection bias and the estimate of VE in reducing severity could be underestimated [15].

As our study is based on a case–case analysis of patients hospitalized with severe laboratory-confirmed influenza, the vaccination effect should be attributed to the capacity to reduce severity after influenza infection had occurred. We do not know through which mechanisms vaccination prevents severity. While protection against influenza infection is primarily mediated by inducing antibodies, protection against severe influenza-related outcomes is mediated through the cellular immune responses affecting viral clearance [25,26]. Unlike antibodies, induced by rapidly mutating surface proteins, cell-mediated immunity to influenza is primarily induced by the major internal virus proteins that are generally more conserved across subtypes, allowing for greater heterologous cross-reactivity [27]. It is suggested that individuals previously infected by seasonal human influenza A viruses or who received seasonal human influenza vaccines may derive benefits, at least in part, from the pre-existing cross-reactive memory of cytotoxic T lymphocytes in reducing the severity of A(H1N1)pdm infection, even without protective antibodies [25].

There were some mismatching seasons (2014/15 for influenza A virus and seasons 2011/12, 2013/14 and 2015/16 for influenza B virus) that may have had some influence on influenza VE.

### Strengths and limitations

The strengths of the study include the large number of patients hospitalised for influenza, the multicentre design, uniform patient screening by hospitals, diagnostic confirmation of all patients and the extended study period of six consecutive influenza seasons (2010/11–2015/16).

The study also had some limitations. First, individuals aged 65 years and older may be less likely to be admitted to the ICU [28], which may have reduced the number of outcomes and the statistical power of the study. Second, although we included a number of potential confounders in the multivariate model, there might be unmeasured confounders associated with vaccination and severe influenza [29]. Third, as the likelihood of ICU admission was greater in those aged 64 and younger and the risk of death was greater in those aged 65 years and older, grouping of patients by these outcomes and age may have underestimated VE. For this reason, we also estimated the influenza VE in those aged 65 years and older, which was higher (25%) than in people aged 18–64 years (13%). Other studies have applied this grouping of death and ICU admission as an outcome of influenza-associated disease severity [21,23]. Finally, physicians may have been more likely to test for influenza virus in patients presenting with more-severe ILI, underestimating the benefit of vaccination. However, as physicians were not aware of the vaccination status of patients it is unlikely that influenza vaccination produced a sampling bias. We only assessed in-hospital deaths we were unable to include deaths that occurred after discharge from hospital.

In conclusion, most patients hospitalised for severe influenza are aged 65 years and older and have underlying medical conditions, leading to a higher risk of influenza-associated complications, ICU admission and death. Influenza vaccination could reduce ICU admission and death in these patients, by reducing the severity of the disease. This effect complements the effectiveness of influenza vaccination in avoiding infection, especially in patients with underlying conditions, in whom influenza vaccination is frequently not optimal in preventing infection. Almost all the patients in this study qualified for influenza vaccination according to national guidelines [30]. Increased vaccination uptake might reduce the number of complications and ICU admissions. Annual influenza vaccination is still the best measure against influenza virus infection and its complications, particularly for populations at risk of more severe disease.

## Notes

**The Working Group on Surveillance of Severe Influenza Hospitalised Cases in Catalonia:** Alsedà M, Álvarez J, Arias C, Balañà PJ, Barrabeig I, Camps N, Carol M, Ferràs J, Ferrús G, Follia N, Godoy P, Bach P, Jané M, Martínez A, Minguell S, Parrón I, Plasència E, Sala-Farré MR, Torner N, Torra R, Torres J (Public Health Agency of Catalonia); Caylà J, Gorrindo P, Rius C (Public Health Agency of Barcelona); Marcos MA, Mosquera MDM, Vilella A (H Clínic, Barcelona); Antón A, Pumarola T, Campins M (H Universitari Vall d’Hebrón, Barcelona); García D (H Josep Trueta, Girona); Espejo E (H Terrassa); Freixas N, Riera Garcia M (Mútua Terrassa); Maraver E, Mas D, Perez R, (H Altahia Manresa); Rebull J (H.Verge de la Cinta, Tortosa); Pou J (H Sant Joan de Déu, Esplugues); García-Pardo G, Olona M (H Joan XXIII, Tarragona); Barcenilla F, Castellana D (H Arnau de Vilanova, Lleida) Navarro-Rubio G (Consorci Sanitari Parc Taulí, Sabadell); Force LL (H Mataró).
